# Whole genome discovery of regulatory genes responsible for the response of chicken to heat stress

**DOI:** 10.1038/s41598-024-56757-0

**Published:** 2024-03-19

**Authors:** Sevda Hosseinzadeh, Karim Hasanpur

**Affiliations:** Department of Animal Science, Faculty of Agriculture, University of Tabriz, Tabriz, Iran

**Keywords:** Long-noncoding-RNAs, Meta-analysis, Acute heat stress, RNA-seq, Genetics, Animal breeding

## Abstract

Long noncoding RNAs (lncRNAs) are functional bridges connecting the genome with phenotypes by interacting with DNA, mRNA, and proteins. Using publically available acute heat stress (AHS)-related RNA-seq data, we discovered novel lncRNAs and tested their association with AHS along with ~ 8800 known lncRNAs and ~ 28,000 mRNA transcripts. Our pipeline discovered a total of 145 potentially novel-lncRNAs. One of them (Fishcomb_p-value = 0.06) along with another novel transcript (annotated as protein-coding; Fishcomb_p-value = 0.03) were identified as significantly associated with AHS. We found five known-lncRNAs and 134 mRNAs transcripts that were significantly associated with AHS. Four novel lncRNAs interact cis-regulated with 12 mRNA transcripts and are targeted by 11 miRNAs. Also six meta-lncRNAs associate with 134 meta-mRNAs through trans-acting co-expression, each targeted by 15 and 216 miRNAs, respectively. Three of the known-lncRNAs significantly co-expressed with almost 97 of the significant mRNAs (Pearson correlation p-value < 0.05). We report the mentioned three known-lncRNAs (ENSGALT00000099876, ENSGALT00000107573, and ENSGALT00000106323) as the most, significantly regulatory elements of AHS in chicken. It can be concluded that in order to alleviate the adverse effects of AHS on chicken, the manipulation of the three regulatory lncRNAs could lead to a more desirable result than the manipulation of the most significant mRNAs.

## Introduction

Heat stress (HS) in animals is usually happened due to the imbalance between the metabolic heat production of the animal and its capacity to dissipate the heat to the surrounding. In birds, especially the commercial chicken strains in high stocking farms in warm areas, due to the absence of sweat glands and the higher metabolic activity of the genetically improved birds, heat stress is now considered as a main concern^1^. When chickens are exposed to high ambient temperatures, several physiological processes are modified in their bodies; including immune function^2^, metabolism^3^, blood flow^2^, cellular processes, oxidative metabolism^4,5^, cell cycle^6^, membrane structure and function^7^, transcription^8^, translation^9^, post-translational modification^10^, Deoxyribonucleic acid (DNA) repair^11^, and protein structure, protein binding, protein translocation and protein formation^12^. Phenotypically, HS in chicken may lead to a suppressed appetite and reduced feed consumption which, in turn, may result in reduced productivity and increased morbidity or mortality^13^ and, ultimately, significant financial losses^14^. As a major organ of metabolism and detoxification in live animals, liver plays a key role in the absorption, distribution and excretion of nutrients^15^. It is worth to note that HS is the main cause of liver damage through the excessive production of reactive oxygen species^16,17^, decreased activity of glutamine synthetase and microvesicular steatosis^18^, impaired hepatic mitochondrial respiration and lipid peroxidation, and excessive formation of malondialdehybe^19^.

Recently, numerous researches have reported multiple small and long non-coding RNAs that are crucially important for the resistance of chicken to HS via altering or regulating the expression levels of the protein coding genes^20–24^. lncRNAs are non-coding RNAs longer than 200 nucleotides, which can be divided into four categories including; intronic, intergenic, sense, and antisense lncRNAs^25^. They have comparably lower conservation scores than the coding genes^26^. In addition, lncRNAs have different biological functions, such as tissue development, cell transport, metabolic and biological processes^27,28^. lncRNAs regulate different stages of mRNA life (e.g., splicing, turnover and translation). Having exons, introns, 3'-polyadenylation, 5'-capping by 7-methylguanosine^29^, and RNA splicing, lncRNAs share many structural or feature similarities to the mRNAs. However, they have little or no open reading frame (ORF) containing fewer than 300 nucleotides or 100 codons^30,31^. miRNAs, on the contrast, are small and single-stranded 18–25 nucleotide non-coding RNAs that regulate many biochemical and physiological processes^32^ such as apoptosis, differentiation, and proliferation^33^. miRNAs also play critical roles in regulating gene expression at the post-transcriptional level through mRNA degradation or translational repression^34^. Recent studies have shown that miRNAs are also principally important for the regulation of lncRNAs and mRNAs^35^.

Generally, HS can induce modifications in gene expression by affecting the expression of lncRNAs and miRNAs. Therefore, the aim of the current work was to identify the association of protein-coding RNAs and lncRNAs with AHS, cyclic temperature about 35 °C and a relative humidity of 60 to 85 percent for 2, 3, 4, 6 or 8 h, and to introduce novel lncRNAs and their contribution to the genome-wide regulation of genes including coding- and non-coding genes.

In this study, we first identified novel lncRNAs and then performed a meta-analysis of the transcriptome to identify key genes and lncRNAs associated with AHS. The results of the meta- analysis were combined with miRNAs to examine the moderating effect of the comprehensive association with AHS. As manipulation of lncRNAs and regulatory miRNAs can lead to more desirable results than manipulation of key mRNAs.

## Material and methods

All of the experimental procedures in the current study were approved by the ethics committee of the Department of Animal Science, University of Tabriz, Iran. The used data were publically available and we did not generate data ourselves.

### Data collection

**Figure 1:**
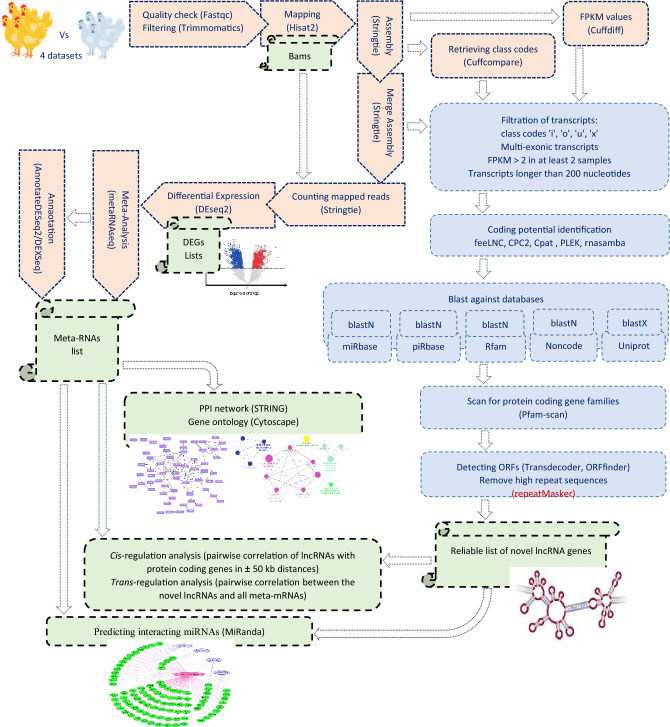
The analytical workflow of the four RNA-seq datasets.

A total of 32 samples belonging to four separate publically available RNA-seq datasets were acquired from the National Center for Biotechnology Information (https://www.ncbi.nlm.nih.gov/sra) by searching for the keywords "chicken," "liver," and "acute heat stress." The datasets were originated to the liver transcriptome data of two layer breeds (i.e., Leghorn, Fayoumi) and one broiler strain which reared under either normal or AHS for 3–4 h conditions^36,37^. A thorough, detailed information on the 32 used RNA-seq samples can be found in Table 1. For the analyses of the four datasets we followed a complex workflow that was illustrated in Fig. 1.

**Table 1:** Accession numbers and meta-data of the used RNA-seq data for the analyses.

Group	Run accession	Raw reads	Alignment rate	Breed	Sex	Age (week)	Duration of heat stress	Sequence type (PE/SE)
	Dataset1							
Case	SRR12073757	47,290,916	88.47	Leghorn	Not collected	2	4 h	51–100 (PE)
	SRR12073758	40,260,177	90.97					
	SRR12073746	41,415,907	91.57					
	SRR12073735	35,611,146	89.07					
Control	SRR12073729	39,001,742	89.45					
	SRR12073730	47,729,079	90.94					
	SRR12073731	52,258,461	90.28					
	SRR12073732	37,009,786	90.31					
Case	Dataset2			Fayoumi	Not collected	2	4 h	54–100 (PE)
	SRR12073747	40,234,382	89.15					
	SRR12073748	44,127,434	90.03					
	SRR12073749	44,711,995	89.72					
	SRR12073750	40,856,455	88.63					
Control	SRR12073742	36,455,690	90.97					
	SRR12073743	22,254,064	91.00					
	SRR12073744	43,734,081	91.18					
	SRR12073745	40,586,395	90.94					
	Dataset3							
Case	ERR1328529	32,837,420	93.80	Broiler	Male	3	3 h	35–100 (SE)
	ERR1328530	27,335,951	94.88					
	ERR1328531	33,532,021	93.91					
	ERR1328532	32,813,911	92.86					
Control	ERR1328525	29,513,248	94.45					
	ERR1328526	30,196,233	93.05					
	ERR1328527	28,977,434	95.49					
	ERR1328528	20,135,467	92.44					
	Dataset4							
Case	ERR1328545	27,065,705	93.80	Fayoumi	Male	3	3 h	42–100 (SE)
	ERR1328546	26,448,275	92.47					
	ERR1328547	31,900,734	91.43					
	ERR1328548	28,297,899	93.87					
Control	ERR1328541	29,571,743	93.18					
	ERR1328542	32,907,381	93.96					
	ERR1328543	35,802,997	94.04					
	ERR1328544	26,358,318	94.27

### Assembly of transcripts and identification of novel lncRNAs

We used the fastq-dump tool of the SRA toolkit (version 2.9.6)^38^ for conversion of SRA files to FASTQ format, the fastQC tool (version 0.12.1)^39^ for quality control of the data, the Trimmomatic software (Version 0.39) ^40^ for trimming-out the low-quality data with ILLUMINACLIP, SLIDING WINDOW (3–5: 20–28), CROP (3–10), AVGQUAL (20–25) and MINLEN (40–45) options, the HISAT2 software (Version 2.2.1)^41^ for mapping the trimmed data onto the reference genome Gallus_gallus.GRCg6a (https://asia.ensembl.org/Gallus_gallus/info/index), and Stringtie (version 1.3.3b)^42^ for both assembling and merging the transcripts. Cuffdiff (Version 2.2.1) and cuffcompare (Version 2.2.1)^43^, were used to calculate the Fragments Per Kilobase of transcript per Million mapped reads (FPKM) values and their corresponding class codes of the expressed transcripts, respectively. Then, a comprehensive filtering pipeline was applied to identify the novel lncRNAs across all the assembled transcripts via excluding of the previously known transcripts, as follow: (1) multi-exonic transcripts longer than 200 nucleotides with class codes 'i', 'o', 'u', 'x' and with FPKM > 2 in at least 2 samples were selected for discovering the potentially novel lncRNA candidates. (2) Five coding-potential-detection software including feeLNC (default parameters)^44^, CPC2 (score > 0.5)^45^, Cpat (score > 0.36)^46^, PLEK (score > 0)^47^, and rnasamba (classification = noncoding) (https://rnasamba.lge.ibi.unicamp.br), were employed and transcripts which identified as coding with at least one of them were excluded. (3) The transcripts were blasted (BLASTn) against the miRbase, piRBase, Rfam, and NONCODE database for removing the transcripts significantly (E-value, 1 × 10^–3^) similar to the known miRNA, piRNA, non-coding RNA families, and noncoding sequences, respectively. (4) Another blast (BLASTx) was conducted on the UniprotKB to remove the transcripts belonging to the known protein coding genes (E-value, 1 × 10^–3^) which was followed by Pfam-scan using the default parameters. (5) TransDecoder tool (version 5.5.0)^48^ and ORFfinder database (https://www.ncbi.nlm.nih.gov/orffinder/) were employed for the prediction of the ORF of the transcripts, and those with ORF longer than 300 (nt) were excluded. RepeatMasker was used to detect and exclude the transcripts with more than expected repeats. In addition to the above mentioned blasts which employ NR database, we applied another BLASTn in NCBI with RefSeq database to filter out the transcripts significantly overlap to the known protein-coding transcripts. Consequently, the transcripts passing the abovementioned filters were categorized in four classes as intergenic, intronic, generic exonic overlap with a reference transcript, and exonic overlap with a reference sequence on the opposite strand. We named the past transcripts (n = 145) as potentially novel-lncRNAs.

### Evaluation of conservation of the identified novel-lncRNAs

Protein-coding genes are known to be highly conserved as compared to the lncRNAs, especially in exonic positions. Therefore, conservation scores of ten randomly chosen protein-coding genes, ten known-lncRNAs, and ten novel-lncRNAs were retrieved from the UCSC Genome Browser (https://genome.ucsc.edu) in which the conservation scores have already been estimated based on the comparison of 77 vertebrates including chicken. Only the conservation scores of the nucleotides located in the exons were taken into account. Average conservation score was estimated for each gene and an ANOVA test was applied to compare the conservation scores among the novel-lncRNA, known-lncRNA, and protein-coding genes, afterward.

### Predicting the regulatory role of novel-lncRNAs on their neighbor protein-coding genes

lncRNAs are well known to have regulating effect on the expression of the protein coding genes via the suppression or activation of transcription in the nearest neighborhood as *cis-*regulatory elements or in not-necessarily close loci as *trans-*regulatory elements^49,50^. Therefore, the protein-coding genes locating ± 50 kb upstream or downstream of each of the 145 novel-lncRNAs were retrieved and the *cis-regulated* target genes were identified. Here, to ensure the identification of non-false positive *cis-*regulatory elements, we went a further filtering step and removed lncRNAs with FPKM = 0 in more than 10 percent of the samples. The co-expression of the novel-lncRNAs and their neighbor protein-coding genes was assessed by Pearson correlation coefficient via ‘correlation’ package in R. The candidate *cis-*regulated target genes for each novel-lncRNA was identified based on (r > =|0.70| and P-value < 0.05).

### Identification of regulatory miRNAs of novel-lncRNAs and their cis-regulated target genes

To identify the miRNAs regulating the lncRNAs and their *cis-*regulated target genes MiRanda software (http://www.microrna.org/microrna/home.do) was utilized with score > 140 and energy < −30 (kcal/mol). Then, we characterized the miRNAs that regulated both the novel-lncRNAs and their *cis-regulated* target genes. It is noteworthy that the mentioned novel-lncRNAs and their cis- regulated target genes had a significant correlation with each other. Finally, the structure of the novel-lncRNAs was characterized employing the TBTools software (version 1.116)^51^.

### Differential expression of all transcripts and meta-analysis

The four datasets were screened separately for differentially expressed transcripts (DETs) between the liver of chickens raised under AHS or normal conditions. All mRNA transcripts (n =  ~ 28,000) and lncRNAs (both known; n =  ~ 8800 and novel; n = 145) were analysed, simultaneously using the DESeq2 package^52^ with the default parameters. AnnotateDESeq2/DEXSeq in usegalaxy (available at https://usegalaxy.eu/) was used to annotate the DESeq2 results to characterize the gene symbols, biotypes, and positions of the transcripts. Subsequently, the metaRNASeq package (version 1.0.2)^53^ in R was used to identify meta- differential transcripts (meta-DETs) by combining p-values via the Fishcomb function. All mRNA and lncRNA transcripts with consistent expression across all datasets (i.e., with positive sign of log2-fold change values in all datasets for consistently up-regulated transcripts and with negative sign of log2-fold change values in all datasets for consistently down-regulated transcripts), and transcripts with Fishcomb p-values < 0.05 were considered as meta-DETs. We named the known mRNA, known-lncRNA and novel-lncRNA meta-DETs as meta-mRNA, meta-lncRNA and meta-novel-lncRNA transcripts, respectively.

### Functional analysis of the meta-coding genes

Protein–protein interaction (PPI) network analysis was done using the STRING database (https://string-db.org/) to create the network and identify the hub-genes. Additionally, ClueGO plugin^54^ of Cytoscape software^55^ was used for gene ontology (GO) and KEGG pathway enrichment analysis. GO terms and pathways with Bonferroni corrected p-values < 0.05 were considered as significantly enriched.

### Co-expression analysis of meta-lncRNA, meta-novel-lncRNA and meta-mRNA transcripts

Identifying the correlated expression between the meta-lncRNAs and meta-mRNAs might facilitate the discovery of the association between the regulatory genes and their regulated genes. To this end, we analyzed the co-expression of the two significant gene biotypes related to AHS (i.e., meta-lncRNAs, meta-novel-lncRNAs with meta-mRNAs) as above. Pearson correlation coefficient values with p-value < 0.05 were introduced as significant. Thereafter, the significant PPI network of those meta-mRNAs that were significantly co-expressed with the meta-lncRNAs was visualized using cytoscape software.

### Prediction of the miRNAs regulating the meta-lncRNA and meta-mRNAs

As mentioned above, MiRanda (v3.3a) was used to predict the miRNAs that more likely regulate the meta-lncRNAs and meta-mRNAs in a two-step analysis based on a score > 140 and Energy < -30 kcal/mol.

### Ethics approval and consent to participate

All of the experimental procedures involving animals used in this study were approved by the Animal Ethics Committee of the Department of Animal Science, University of Tabriz, Iran (Permission number: 955/2022). We have complied with ARRIVE at submission.

## Results

### Identification of novel-lncRNAs

**Figure 2:**
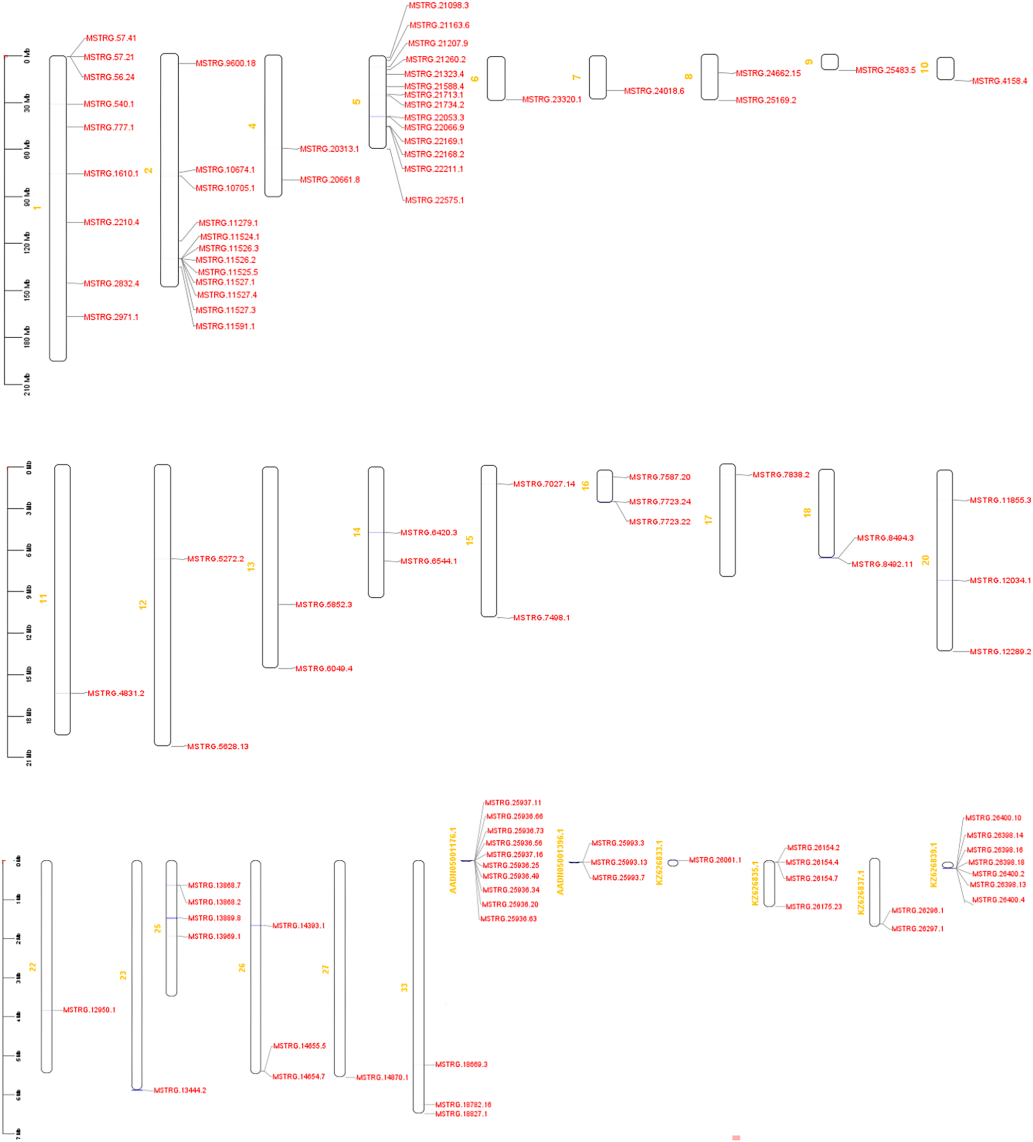
Genome wide distribution of the discovered novel-lncRNAs.

After mapping the trimmed RNA-seq data onto the reference genome and combining all assemblies of transcripts, a total of 106,731 transcripts including 39,280 known transcripts (28,344 protein coding, 8867 lncRNAs) and 67,450 unknown transcripts were identified. Out of the 67,450 unknown transcripts, a total of 4508, 1355, 5622, and 3519 transcripts were classified with class codes 'u' (intronic), 'i' (intergenic), 'o' (generic exonic overlap with a reference transcript), and 'x' (exonic overlap with a reference sequence on the opposite strand), respectively. Results of the filtering pipeline to identify novel-lncRNAs were as follows: 1) Only 6059 transcripts based on FPKM > 2 in at least 2 samples remained. 2) 5766 of them were multi-exonic with length longer than 200 nucleotides. 3) Based on the coding potential results using software such as feeLNC, CPC2, Cpat, PLEK, and rnasamba, a total of 4448 transcripts showed coding potential and thus were excluded and 1318 transcripts were remained. 4) Blasting against the miRbase, piRBase, Rfam, NONCODE, UniprotKB databases, a total of 242, 207, 48, 276, 130 transcripts were filtered out, respectively. Additional, filtering by Pfam-scan, repeatMasker, TransDecoder plus ORF finder, and BLAST with RefSeq database removed 1, 94, 104 and 71 transcripts, respectively. Finally, 145 potentially novel-lncRNA transcripts were identified with 20, 62, 20, and 43 transcripts belonging to the classes of u, i, o, and x, respectively. Most of the novel-lncRNAs located on chromosome 1. At least 15 loci had more than one lncRNA transcripts (ranged 2–8; totally 48) while the remaining 97 loci had only one transcript each. A detailed information about the novel-lncRNAs is provided in Supplementary File 1. While the genetic map of the identified novel-lncRNAs are shown in Fig. 2.

**Figure 3:**
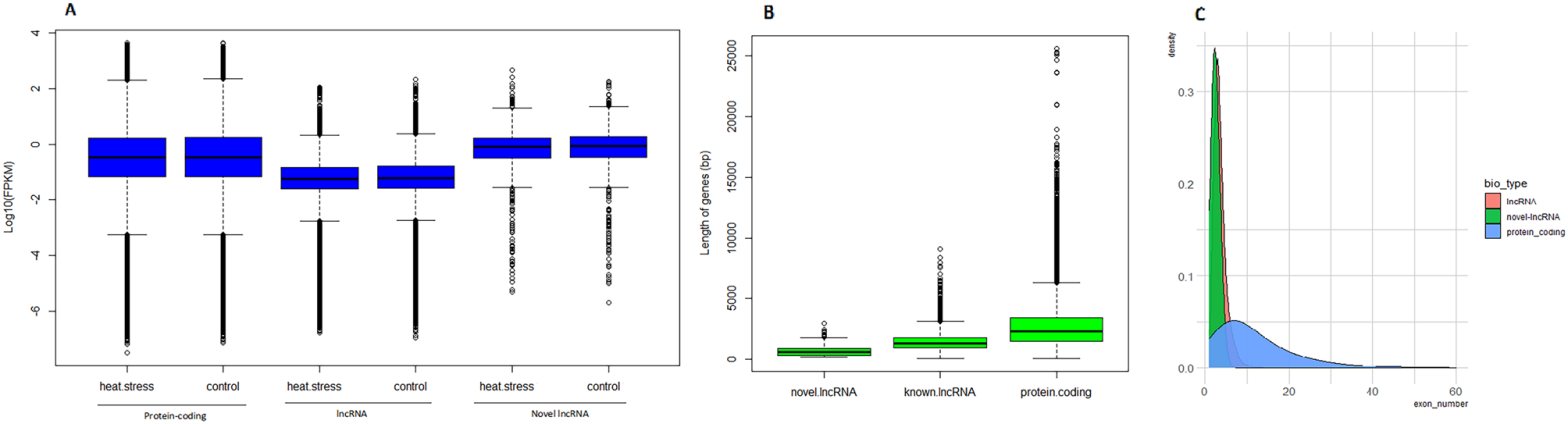
Basic features of the identified 145 novel-lncRNAs in comparison with those of the known-lncRNAs and mRNAs. (**A**) Expression values (log10-FPKMs) of the transcripts in the liver of the heat stressed and un-challenged control chickens. (**B**) Length distribution of the transcripts (base pair) C. Density plot of the number of exons per transcript.

The expression level of the 145 novel-lncRNAs, ~ 8800 known-lncRNAs, and all mRNAs are illustrated in Fig. 3A. The length (bp) and exon number of the 145 novel-lncRNAs were compared with those of the known-lncRNAs, and mRNAs. The resulted plots are shown in Fig. 3B, C. Similar to those of the known lncRNAs, the number of exon of the novel-lncRNAs ranged from 2 to 5 exons, while the number of exon of the mRNAs ranged more widely, with some transcripts having more than 60 exons (Fig. 3C).

### Co-expression of novel-lncRNAs and mRNAs

*Cis-*regulation of the protein coding genes by the novel-lncRNAs were calculated using the co-expression analysis. As a result, 12 significant (p-value < 0.05) correlations were identified between the 4 novel-lncRNAs and 12 mRNA transcripts. Three out of the 12 significant correlations were negative while the remaining nine were positive. Three novel-lncRNAs correlated with more than one mRNA each. For instance, MSTRG.1100.2 significantly co-expressed with six protein coding genes including RAC2, IL2RB, MPST, TST, C1QTNF6 and TMPRSS6, while MSTRG.19559.1 significantly co-expressed with three protein coding genes including COMMD5, LONRF3, and FAM199X, and MSTRG.25483.5 significantly co-expressed with two protein coding genes including TFDP2, and ATP1B3. In contrast, MSTRG.57.21 significantly co-expressed with only one protein coding gene named CALU (Supplementary Fig. 1).

**Figure 4:**
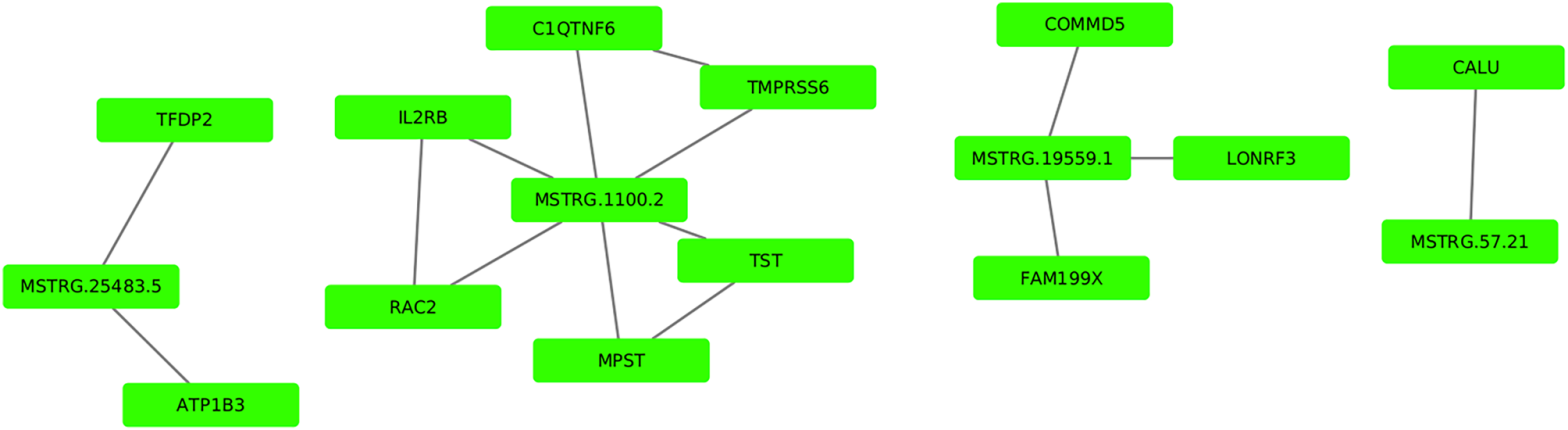
Protein–protein interaction (PPI) network analysis of the protein coding genes targeted by the novel-lncRNAs.

Based on the PPI network analysis of the 12 *cis-*regulated target genes using the STRING we found superficial relationships among some of the target protein coding genes. Some of the relationships were based on only two genes. For instance, TST related to MPST, IL2RB related to RAC2, and TMPRSS6 related to C1QTNF6 and made simple networks. Surprisingly, adding the novel-lncRNAs to the network of their target protein coding genes created bridges between the simple networks and linked the simple networks each other and made a complex network. In Fig. 4 the complex relationship among the novel-lncRNAs and their target protein coding genes is illustrated.

**Figure 5:**
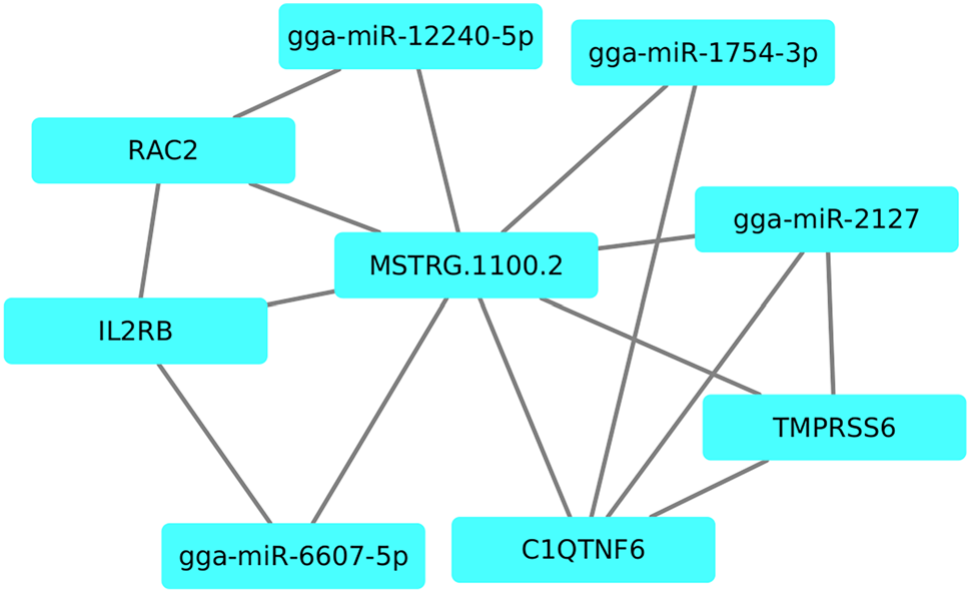
Protein–protein interaction (PPI) network analysis of the protein-coding genes targeted by the novel-lncRNAs and miRNAs.

In addition to the association of lncRNAs and their neighbor protein-coding genes, we predicted the miRNAs that target both novel-lncRNAs and the 12 cis-regulated protein-coding genes in two separate analysis using the MiRanda. A total of 4 novel-lncRNAs were predicted to be targeted by 11 miRNAs. Moreover, the 12 cis-regulated protein-coding genes were predicted to be targeted by 128 miRNAs (Supplementary Fig. 2). Four of the miRNAs (i.e., gga-miR-6607-5p, gga-miR-2127, gga-miR-12240-5p, and gga-miR-1754-3p) targeted a novel-lncRNAs (MSTRG.1100.2) and its 4 *cis-regulated* protein-coding genes (IL2RB, TMPRSS6, RAC2, and C1QTNF6), simultaneously (Fig. 5).

### Conservation analysis

**Figure 6:**
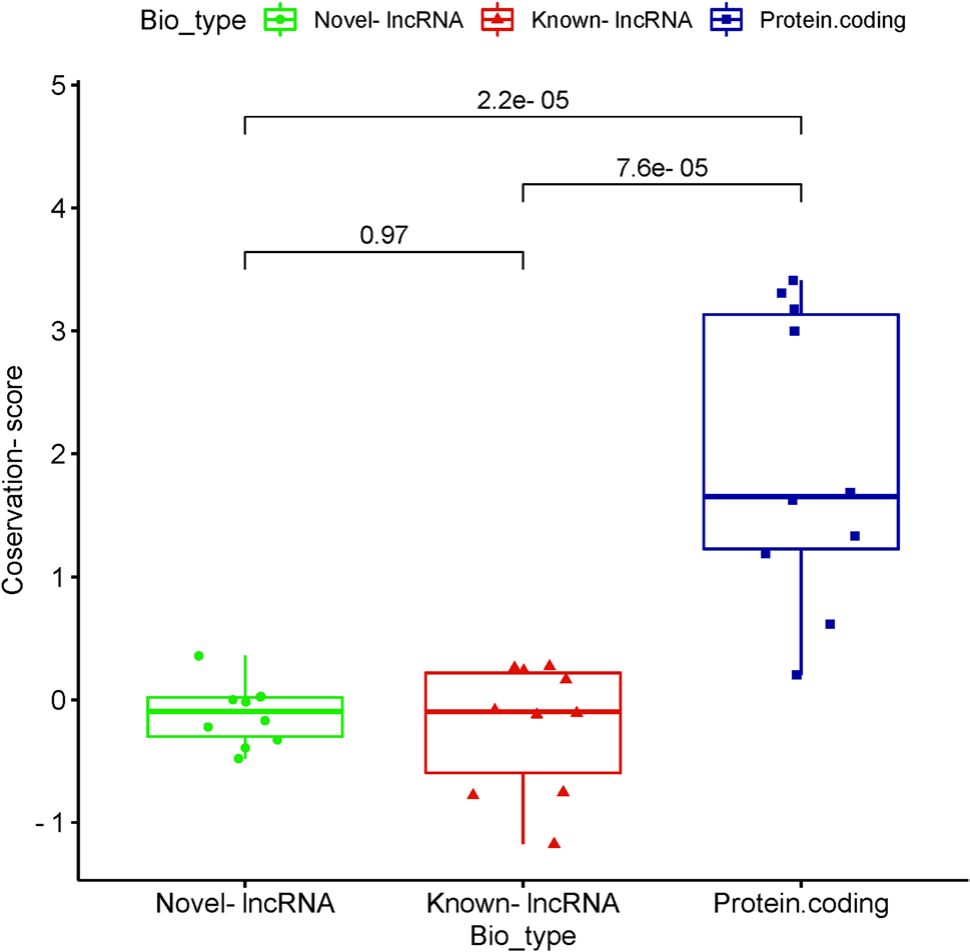
Comparison of conservation score of novel-lncRNAs, known-lncRNAs, and protein coding transcripts. The comparison was made based on the average conservation score of exonic locations of ten transcripts within each biotype.

Based on the ANOVA test and pairwise comparing the average conservation scores of novel-lncRNAs, known-lncRNAs, and mRNAs, a statistically significant differences was observed between the novel-lncRNAs and mRNAs (p-value = 1.48E−05). There was, however, no significant difference between the novel- and known-lncRNAs (Fig. 6). The thorough information about the ANOVA test of the conservation scores between the lncRNAs and mRNAs are reported in Supplemental File 2.

### Meta-analysis

The differential expression analysis between the AHS-challenged and un-challenged normal chickens in each of the four independent datasets revealed a relatively low number of differentially expressed transcripts (DETs) with limited number of genes shared by two or more datasets. For detailed information about the DETs and overlaps of them in the four datasets see our previous research^12^. Briefly, based on the adjusted p-values < 0.05 a total of 41 (mRNA = 38, lncRNA = 3), 32 (mRNA = 27, lncRNA = 5), 114 (mRNA = 106, lncRNA = 8), and 45 (mRNA = 44, lncRNA = 1) DETs were found for the four datasets (Supplementary File 3).

**Figure 7:**
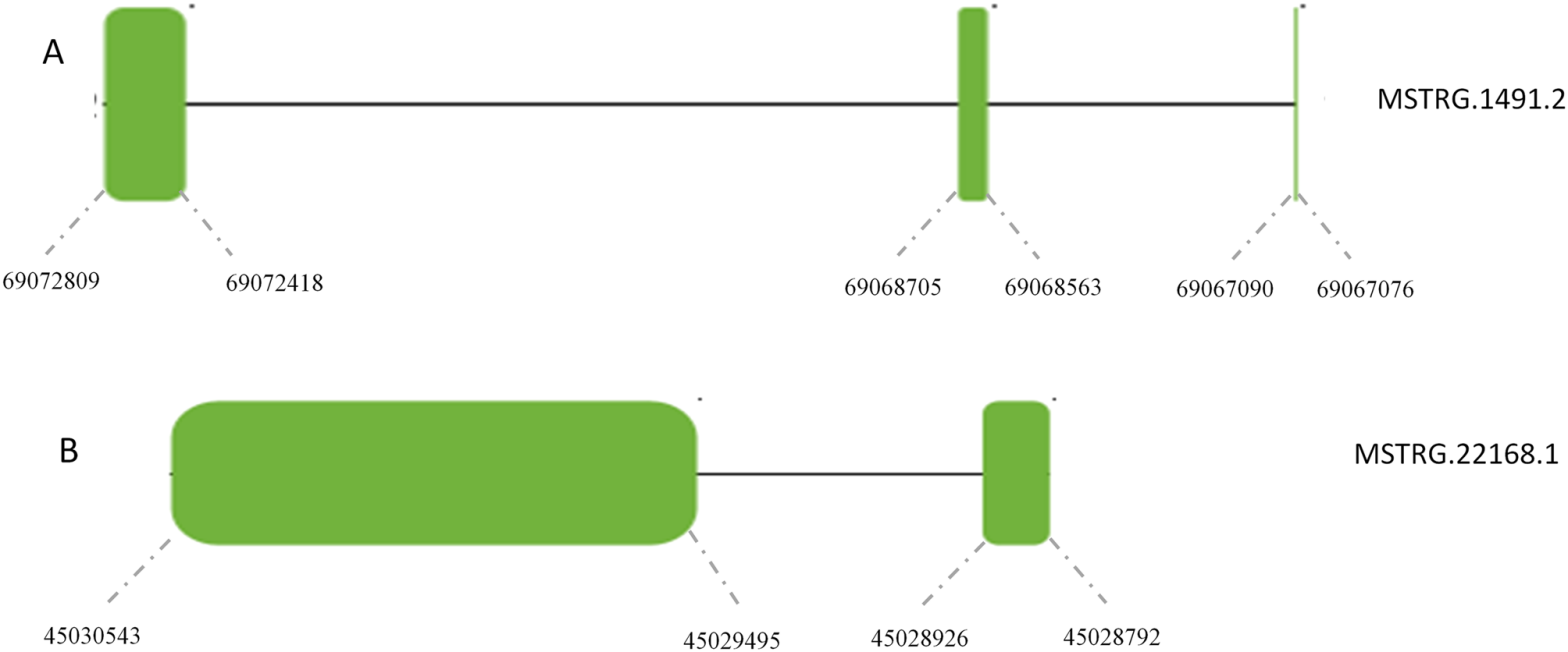
Exon–intron structures of the two identified novel transcripts.

Meta-analyzing of the output of the four datasets by combining their p-values, we could identify 134 mRNA transcripts (78 up-regulated and 56 down-regulated in all data sets). We will call these 134 transcripts as meta-mRNAs. Supplementary File 4 includes a detailed information about the results of meta-analysis on the meta-mRNAs. In addition to the 134 meta-mRNAs we identified five differentially expressed up-regulated known-lncRNAs (will be called as meta-lncRNAs hereafter) as was reported in Table 2. Interestingly, all of the five mentioned meta-lncRNAs were over-expressed under the heat stress condition in all four datasets, indicating an important role of them in alleviating the adverse effect of heat stress from the chicken liver. Additionally, one of the novel-transcripts (i.e., MSTRG.1491.2) revealed to be differentially up-regulated (meta-analysis p-value = 4.90174E−06) between the AHS and normal conditions. It is an intergenic novel-lncRNA with transcript length of 550 base that located on reverse strand of chromosome 1 with three exon spanning on Exon 1: 69,067,076–69,067,090, Exon 2: 69,068,563–69,068,705, and Exon 3: 69,072,418–69,072,809 bp. Screening the ENSEMBL and NCBI genome browser we found out that the same transcript has already been detected elsewhere by other RNA-Seq studies. It shares exons with PNPLA3 transcripts. However, since it passed all of the coding potential identification filtration steps in the current work, we decided to not exclude it from further analysis and aimed to check its regulatory potential as well (Fig. 7A). Additionally, one of the 145 novel-lncRNAs (i.e., MSTRG.22168.1) tend to be differentially down-regulated by the AHS (meta-analysis p-value = 0.067). It is an intergenic lncRNA with transcript length of 1184 base that located on reverse strand of chromosome 5 with two exons spanning on Exon 1: 45,028,792–45,028,926, and Exon 2: 45,029,495–45,030,543 bp **(**Fig. 7B). In Fig. 7 the Exon–Intron structure of the mentioned novel transcript has been shown.

**Table 2:** Differentially expressed known-lncRNAs identified by meta-analysis of four independent datasets.

Transcript ID	log2(FC)	log2(FC)	log2(FC)	log2(FC)	P-value	P-value	P-value	P-value	meta-analysis
ENSGALT00000095990	0.113	4.495	0.513	0.780	0.930	1.54E-05	0.626	0.442	0.001
ENSGALT00000099876	0.026	1.547	1.603	0.207	0.968	0.015	0.035	0.744	0.047
ENSGALT00000106265	1.276	2.103	1.076	1.699	0.139	0.055	0.423	0.091	0.039
ENSGALT00000106323	1.291	0.832	0.609	1.340	0.050	0.249	0.296	0.006	0.006
ENSGALT00000107573	1.089	1.051	1.255	0.635	0.167	0.027	0.135	0.260	0.025

**Figure 8:**
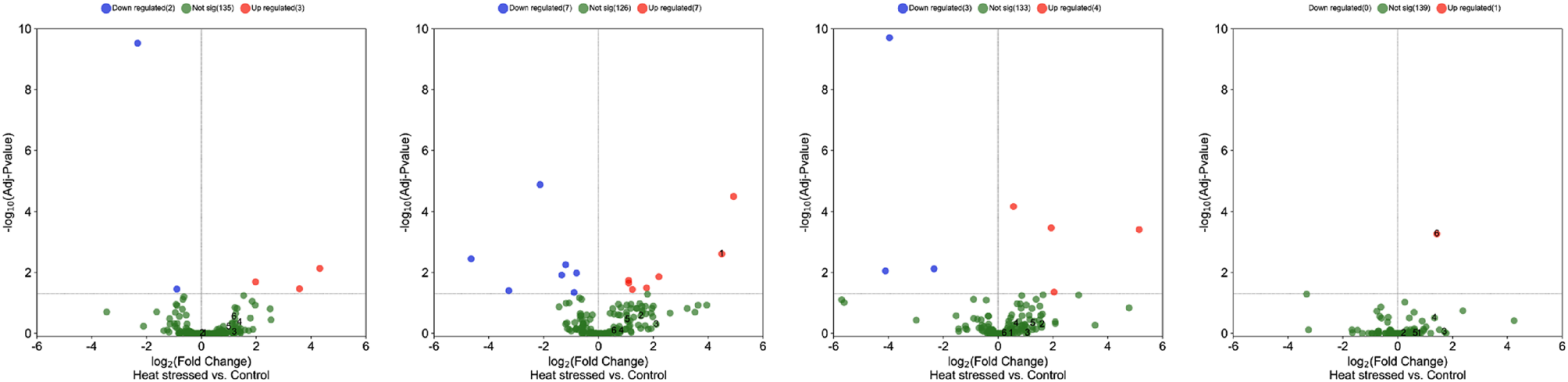
The volcano plot of the expression of the 140 meta-DETs within the four datasets. From the left to right, the datasets were, dataset1, dataset2, dataset3, dataset4, respectively. Here, for simplifying the understanding of the power of meta-analysis in detecting the significant but less variable genes related to the trait of interest, the expression pattern of the differentially expressed transcripts within each of the four datasets were not shown.

In Fig. 8 the volcano plot of the expression of the 140 meta-DETs (134 meta-mRNAs, 5 meta-lncRNA, and 1 meta-novel-lncRNA) in the four datasets are shown. Here, to show the necessity of the implementation of meta-analysis on the multiple similar datasets, we only show the expression pattern of the 140 meta-DETs on the four individual datasets analyses and not all of the detected DEGs in each of them. As can be seen in the volcano plot, almost 90% of the meta-DETs were differentially expressed in neither of the four individual analysis indicating that the implementation of the meta-analysis for the discovery of the differential transcripts with minimal differences, especially the lncRNAs, were critically necessary.

### Functional analysis of the meta-mRNAs

**Figure 9:**
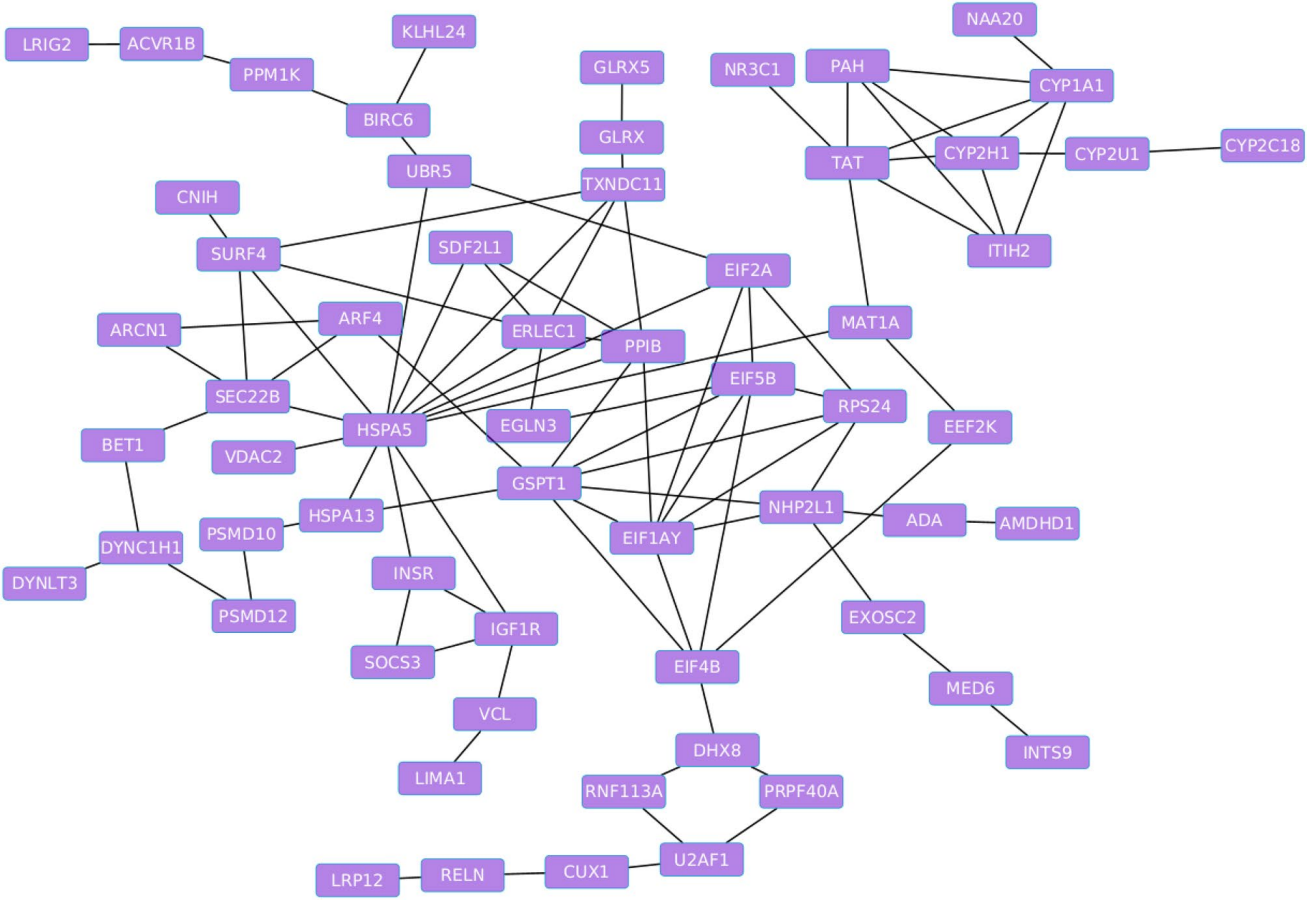
Protein–protein interaction (PPI) network analysis of 134 meta-mRNAs. The significant differential expression of these 134 mRNA transcripts were identified following the meta-analysis of four distinct but similar RNA-seq datasets (Fishcomb p-value < 0.05). All datasets were originated from the liver transcriptome of the chickens that were challenged with acute heat stress (half of the samples) for 3–4 h in order to compare with those of un-challenged control ones.

**Figure 10:**
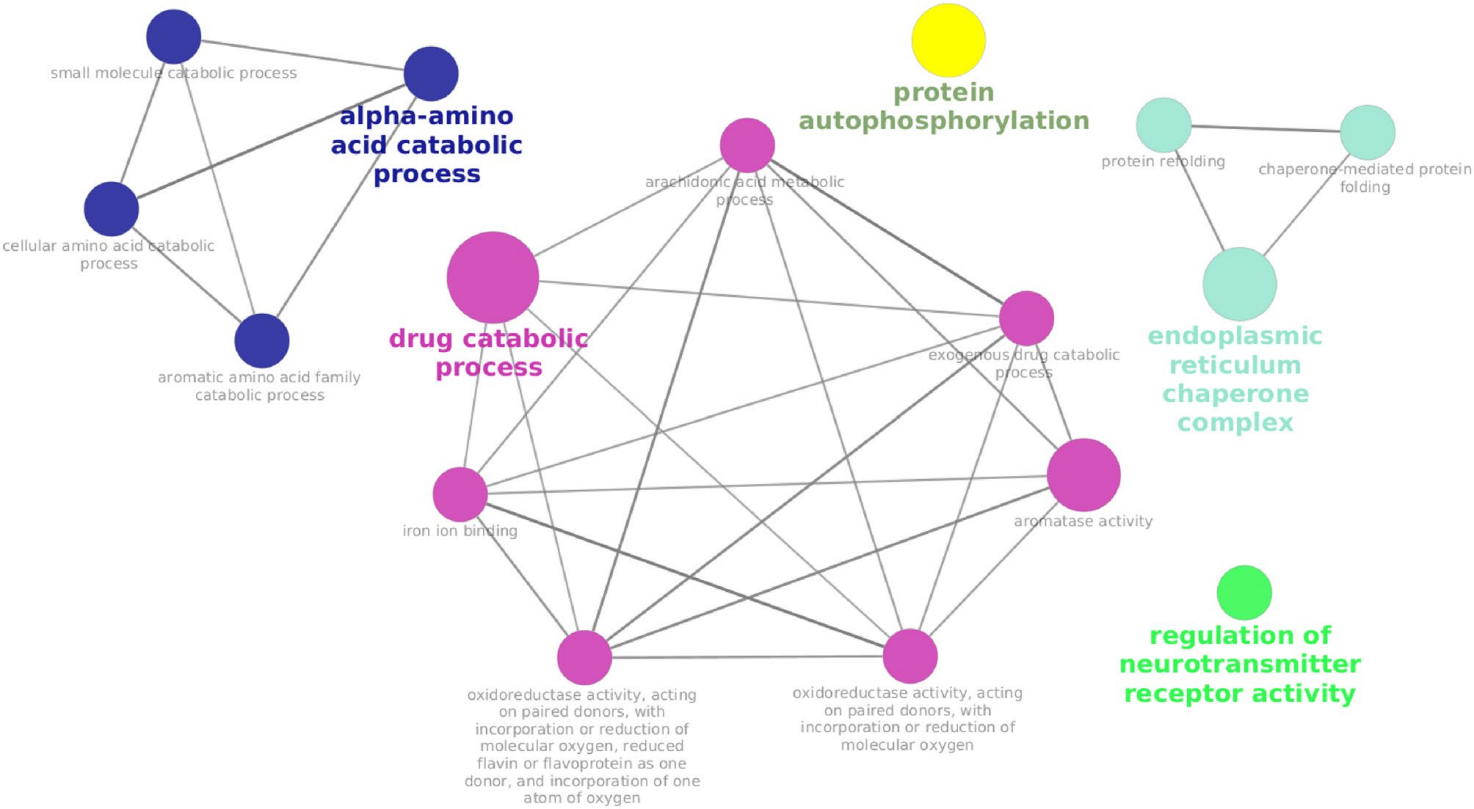
The association of the identified significant gene ontology terms (Bonferroni adjusted p-value < 0.05) enriched by the 134 meta-mRNAs.

After the identification of 134 meta-mRNAs, their PPI network was constructed using STRING as was illustrated in Fig. 9. The constructed network showed considerable edges between the nodes indicating a significant relationship among the meta-mRNAs. Additionally, GO and KEGG pathway analyses were performed by ClueGO plugin of the cytoscape on the 134 meta-mRNAs (Fig. 10). As such, we identified 14, 1, and 1 significant Biological processes (BP), cellular components (CC), and Molecular Function (MF) terms, respectively. The terms “protein autophosphorylation”, “regulation of neurotransmitter receptor activity”, “protein refolding”, and “chaperone-mediated protein folding”, were among the most significantly enriched BP terms while “endoplasmic reticulum chaperone complex”, and “iron ion binding” were significant CC and MF terms, respectively (Supplementary File 5).

### Trans-acting co-expression analysis between the meta-lncRNAs and meta-mRNAs

**Figure 11:**
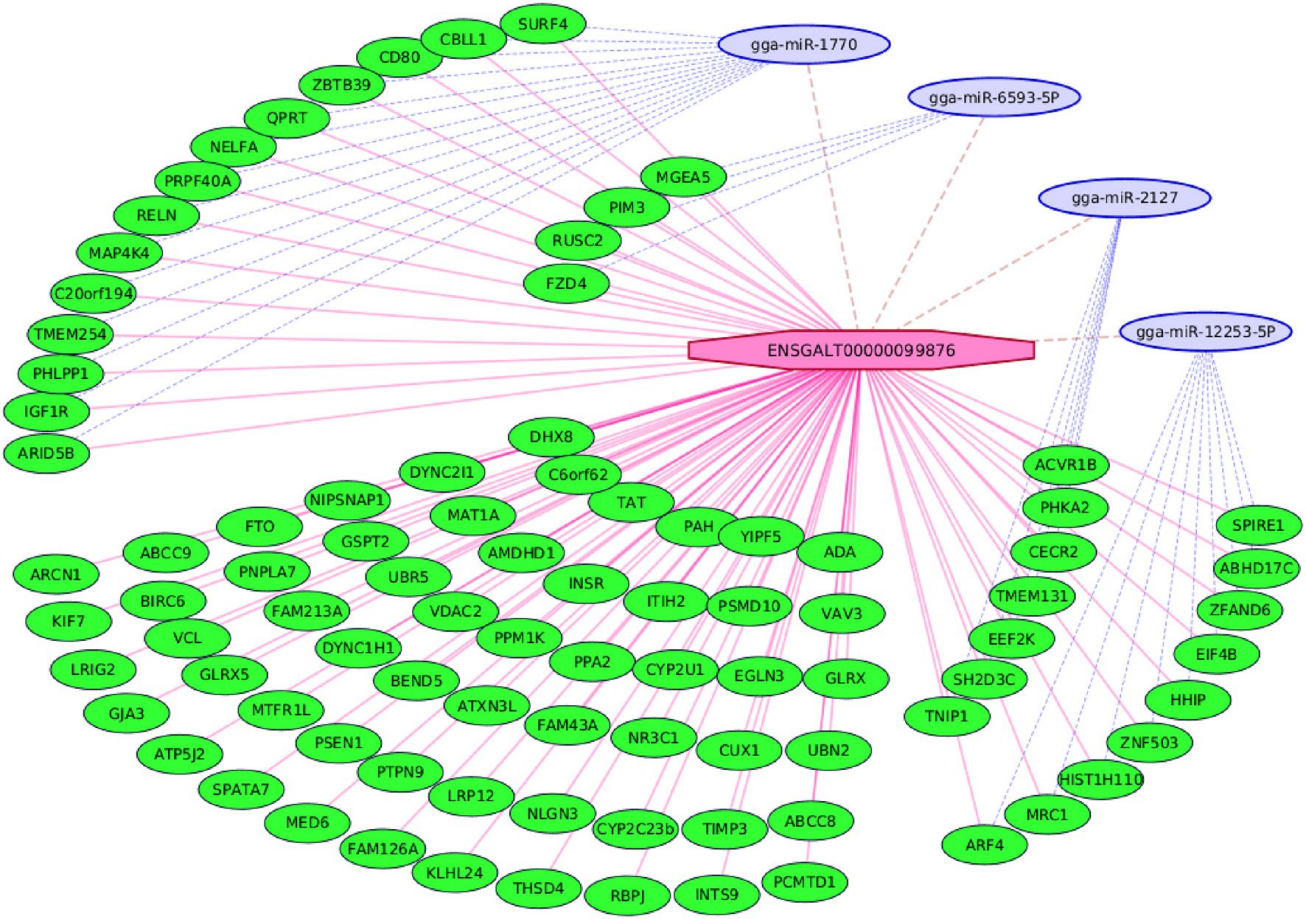
The regulatory networks between the ENSGALT00000099876 meta-lncRNA and its targeted 91 meta-mRNAs (the red edges). In addition, the mutual regulation of 35 meta-mRNAs by ENSGALT00000099876 meta-lncRNA and four miRNAs is illustrated with the blue, dashed edges. Some of the meta-mRNAs were under the regulation of more than one miRNAs, however in this figure only one of them were shown to make the understanding of the network easy. All of the transcripts were significantly differentially expressed between the liver tissue of chickens under acute heat stress and their control counterparts, which identified following the meta-analysis of four distinct but similar RNA-seq datasets (Fishcomb p-value < 0.05).

*Trans*-acting co-expression analysis was carried out here to associate the 6 lncRNAs (5 meta-lncRNAs and 1 meta-novel-lncRNA) with the 134 meta-mRNAs. Correlations with p-values < 0.05 were considered as significant. Remarkably, a large number of significant correlations (n = 97) were identified between 3 meta-lncRNAs and 92 meta-mRNAs. Even more surprisingly, one of them (i.e., ENSGALT00000099876) correlated with almost all of them (i.e., 91 significant co-expressions with correlations ranged 0.35–0.78) indicating that the mentioned known meta-lncRNA undertook the regulation of most of the meta-mRNAs that were in association with AHS. In other words, a small modification in the expression of ENSGALT00000099876 meta-lncRNA may lead to remarkable changes in the expression of tens of protein coding RNAs that are related to AHS and is introduced as a key regulatory gene related to the response of chicken to AHS. ENSGALT00000107573 and ENSGALT00000106323 meta-lncRNAs also showed significant co-expressions with 5 and 1 meta-mRNAs, respectively. Obviously, some of the meta-mRNAs were under the control of two lncRNAs. For example, ABCC8, ABCC9, CYP2C23b and RBPJ meta-mRNAs were under the regulatory control of two meta-lncRNAs including ENSGALT00000107573 and ENSGALT00000099876. In Fig. 11 the regulatory network of ENSGALT00000099876 and its co-expressed protein coding genes is shown. Additionally, in Supplementary File 6 the detailed information of the association of three known meta-lncRNAs and 92 meta-mRNAs related to AHS is reported.

### Prediction of miRNA regulating the meta-lncRNA and meta-coding genes

In this study, 16 miRNAs:meta-lncRNA pairs and 1733 miRNA:meta-mRNA pairs were detected. A total of 216 miRNAs were predicted to regulate the 121 meta-mRNAs and 15 miRNAs were predicted to regulate 5 meta-lncRNAs. The complete information is available in Supplementary File 7. gga-miR-12237-5p, gga-miR-12215-5p, gga-miR-12279-5p, gga-miR 12245-3p, and gga-miR-12282-5p were associated with 101, 73, 71, and 69 meta-mRNAs, respectively. Supplemental Fig. 3 shows the target genes of miRNAs and meta-lncRNAs. In addition, 4 miRNAs (gga-miR-12253-5p, gga-miR-1770, gga-miR-2127, and gga-miR-6593-5p) were predicted to regulate both the ENSGALT00000099876 meta-lncRNA and 35 of its targeted meta-mRNAs. In Fig. 11 the mutual regulatory relationships among the four miRNAs, one meta-lncRNAs and 35 meta-mRNAs are illustrated.

## Discussion

According to the main roles of lncRNA in the regulation of RNA at the epigenetic level, including the regulation of DNA methylation^56^, histone modification^57^ and chromatin remodeling^58^, and at the transcriptional level, including the interaction with transcription factors^59–62^, proteins in the nucleus^63^ and proteins in the cytoplasm^64^, and interaction with miRNAs^22,65–69^. Several studies have shown that the expression of some genes under AHS is modified^36,70–72^, possibly through the interaction with small or long noncoding RNAs^20–24^. Therefore, the identification of key coding genes and their corresponding regulatory elements (i.e., lncRNA and miRNA) is critically important to reveal the causative factors associated with AHS in liver of chickens, as liver is extremely vulnerable under the influence of heat stress^73,74^. Structural similarity of lncRNA to mRNA^27–29^ has allowed us to investigate both of them simultaneously using RNA-seq data. The discovery of the novel transcripts including the novel lncRNAs has also been made more recently using RNA-seq data. We, in the current work, identified 134 meta-mRNAs which more than two third (92 mRNAs) were revealed to be under the regulatory control of three lncRNAs. In addition, almost 90% of them (121 out of the 134 mRNAs) were found to be regulated by 216 miRNAs. According to the previous studies and the findings of the current work, the expression of several important genes are modified under HS; including HSPA5^12,36,72,75,76^, EIF2A^77–79^, RBPJ^80^, ABCC9^81^, TAT^82,83^, ATP5J2^84^, CD80^85^, EEF2K^86^, EIF4B^87^, FTO^88,89^, GLRX^90^, IGF1R^91^, INSR^77,92^, VAV3^93^, UBR5^94^, TNIP1^90^, PTPN9^95^, EIF2A^96^, AKR1D1^97^, HSPA13^98^, CYP1A2^99^, and SDF2L1^12^. Additionally, based on our result, other protein-coding genes such as ABCC9, TAT, ATP5J2, CD80, EEF2K, EIF4B, FTO, GLRX, IGF1R, INSR, VAV3, UBR5, TNIP1, RBPJ, and PTPN9, were significantly modified with the heat stress in conjunction with the regulation effect of ENSGALT00000099876, ENSGALT00000106323, and ENSGALT00000107573 lncRNAs. GO analysis revealed two not well known heat shock proteins including HSPA13 and HSPA5 as important players of the role of “protein refolding” and “chaperone-mediated protein folding”. HSPA5 and SDF2L1 involve in “endoplasmic reticulum chaperone complex” and CYP1A2 play critical role in “oxidoreductase activity, acting on paired donors, with incorporation or reduction of molecular oxygen,” “oxidoreductase activity, acting on paired donors, with incorporation or reduction of molecular oxygen, reduced flavin or flavoprotein as one donor, and incorporation of one atom of oxygen,” “iron ion binding,” and “aromatase activity” processes. Moreover, FTO has significant role in “oxidoreductase activity, acting on paired donors, with incorporation or reduction of molecular oxygen,” and “iron ion binding” processes. Additionally, EEF2K, EIF2A, IGF1R, and INSR genes are known to associate in the “protein autophosphorylation” process.

We also used a computational frame work to identify the regulatory functions of the novel lncRNA in liver of chickens under AHS. There are documented reports that lncRNAs regulate genes which located in their close vicinity^100^. In other words, the function of the lncRNAs can be deduced by analyzing the genes located physically in their proximity. Our results indicated that there are 12 mRNAs transcribing genes at intervals of less than 50 kb upstream or downstream to the four novel lncRNAs. According to the RefSeq report, their roles are as follows; TST involve in 5S-rRNA binding and thiosulfate sulfurtransferase activity^101^, TMPRSS6 involve in matrix remodeling processes in the liver^102^, RAC2 involve in the generation of reactive oxygen species^103^, LONRF3 involve in protein–protein and protein-DNA interactions^104^, CALU involve in ER functions as protein folding and sorting, ATP1B3 involve in osmoregulation, sodium-coupled transport of organic and inorganic molecules, and electrical excitability of muscle and nerve, TFTP2 involve in cell cycle, MPST involve in transfer of a sulfur ion from 3-mercaptopyruvate to thiol compounds, and IL2RB involve in T cell-mediated immune responses play roles. As can be postulated, most of them are those genes that are related to the process of folding the proteins. We in our previous work have found that the acute heat stress deteriorates the process of folding of the proteins in liver^12^.

## Conclusion

The identification or discovery of the previously unidentified genes or gene features has become possible just recently owing to the advances have been made to the technology of RNA sequencing. Comparing the number of identified genes in human or mouse (two more researched species) with that of the domestic animals, it can be postulated that at least hundreds or thousands of genes have been remained to be comprehensively discovered or annotated. We, in the current work, discovered more than one hundred active lncRNAs among the more than 50 thousands assembled unknown transcripts. In total, 145 potentially novel-lncRNA transcripts were found, with 20, 62, 20, and 43 transcripts belonging to the u, i, o, and x classes, respectively. We found 134 mRNA transcripts, 78 up-regulated and 56 down-regulated, in all data sets, and five up-regulated known-lncRNAs. One of the novel-transcripts, MSTRG.1491.2, was significantly up-regulated (p-value = 4.90174E−06) between AHS and normal conditions. 134 meta-mRNAs analyzed by The ClueGO plugin on cytoscape, we came across 16 important terms, including “protein autophosphorylation,” “regulation of neurotransmitter receptor activity,” “protein refolding,” “chaperone-mediated protein folding,” “endoplasmic reticulum chaperone complex,” and “iron ion binding” were significant terms, respectively. Four novel lncRNAs interact cis-regulated with 12 mRNA transcripts and are targeted by 11 miRNAs. Also Six meta-lncRNAs associate with 134 meta-mRNAs through trans-acting co-expression, respectively. Three of the known-lncRNAs significantly co-expressed with almost 97 of the significant mRNAs (Pearson correlation p-value < 0.05). The significantly differentially expressed novel transcripts along with the previously known lncRNAs and mRNAs indicate that the regulatory elements of gene expression are as equally or even more important than the protein coding genes in contribution to the response of the animals to the environmental stressors. For instance, we found a known significantly differentially expressed lncRNA between the AHS-challenged birds and their un-challenged control counterparts (i.e., ENSGALT00000099876) that undertook the regulation of almost 70% (91 out of the 134) of the significantly differentially expressed protein coding genes. We introduce here the mentioned lncRNA as well as the reported 134 protein coding genes as the gene set that are more probably responsible to the compatibility response of the domestic chicken to the acute heat stress.

### Supplementary Information

Supplementary Information 1.

Supplementary Information 2.

Supplementary Information 3.

Supplementary Information 4.

Supplementary Information 5.

Supplementary Information 6.

Supplementary Information 7.

Supplementary Information 8.

## Data Availability

All data supporting the conclusions of this study are included within the article (and additional files). The sequencing data were acquired from the NCBI and we did not carry out the sequencing ourselves.
