# Supplementary material for: Whole genome discovery of regulatory genes responsible for the response of chicken to heat stress
Source: Sci Rep. 2024 Mar 19;14:6544. doi: 10.1038/s41598-024-56757-0 (PMC10951342; doi:10.1038/s41598-024-56757-0)
Supplement: Supplementary file 8 — Supplementary Information 8. [file 41598_2024_56757_MOESM8_ESM.docx]

**Whole genome discovery of regulatory genes responsible for the response of chicken to heat stress**

*Sevda Hosseinzadeh and Karim Hasanpur**

Department of Animal Science, Faculty of Agriculture, University of Tabriz, Tabriz, Iran.

* Corresponding Author E-mail address: [karimhasanpur@yahoo.com](mailto:karimhasanpur@yahoo.com)

| Table S1. Information of the used datasets for the analyses | | | | | | | | |
| --- | --- | --- | --- | --- | --- | --- | --- | --- |
| Group | Run accession | Raw Reads | Alignment Rate | Breed | Sex | Age at Sample Collection (week) | Durtion of heat stress | Sequence length (PE / SE) |
| Case | SRR12073757 | 47290916 | 88.47 | Leghorn | not collected | 2 | 4 hours | 51-100  (PE) |
|  | SRR12073758 | 40260177 | 90.97 |  |  |  |  |  |
|  | SRR12073746 | 41415907 | 91.57 |  |  |  |  |  |
|  | SRR12073735 | 35611146 | 89.07 |  |  |  |  |  |
| Control | SRR12073729 | 39001742 | 89.45 |  |  |  |  |  |
|  | SRR12073730 | 47729079 | 90.94 |  |  |  |  |  |
|  | SRR12073731 | 52258461 | 90.28 |  |  |  |  |  |
|  | SRR12073732 | 37009786 | 90.31 |  |  |  |  |  |
| Case | SRR12073747 | 40234382 | 89.15 | Fayoumi | not collected | 2 | 4 hours | 54-100  (PE) |
|  | SRR12073748 | 44127434 | 90.03 |  |  |  |  |  |
|  | SRR12073749 | 44711995 | 89.72 |  |  |  |  |  |
|  | SRR12073750 | 40856455 | 88.63 |  |  |  |  |  |
| Control | SRR12073742 | 36455690 | 90.97 |  |  |  |  |  |
|  | SRR12073743 | 22254064 | 91.00 |  |  |  |  |  |
|  | SRR12073744 | 43734081 | 91.18 |  |  |  |  |  |
|  | SRR12073745 | 40586395 | 90.94 |  |  |  |  |  |
| Case | ERR1328529 | 32837420 | 93.80 | Broiler | male | 3 | 3 hours | 35-100  (SE) |
|  | ERR1328530 | 27335951 | 94.88 |  |  |  |  |  |
|  | ERR1328531 | 33532021 | 93.91 |  |  |  |  |  |
|  | ERR1328532 | 32813911 | 92.86 |  |  |  |  |  |
| Control | ERR1328525 | 29513248 | 94.45 |  |  |  |  |  |
|  | ERR1328526 | 30196233 | 93.05 |  |  |  |  |  |
|  | ERR1328527 | 28977434 | 95.49 |  |  |  |  |  |
|  | ERR1328528 | 20135467 | 92.44 |  |  |  |  |  |
| Case | ERR1328545 | 27065705 | 93.80 | Fayoumi | male | 3 | 3 hours | 42-100  (SE) |
|  | ERR1328546 | 26448275 | 92.47 |  |  |  |  |  |
|  | ERR1328547 | 31900734 | 91.43 |  |  |  |  |  |
|  | ERR1328548 | 28297899 | 93.87 |  |  |  |  |  |
| Control | ERR1328541 | 29571743 | 93.18 |  |  |  |  |  |
|  | ERR1328542 | 32907381 | 93.96 |  |  |  |  |  |
|  | ERR1328543 | 35802997 | 94.04 |  |  |  |  |  |
|  | ERR1328544 | 26358318 | 94.27 |  |  |  |  |  |


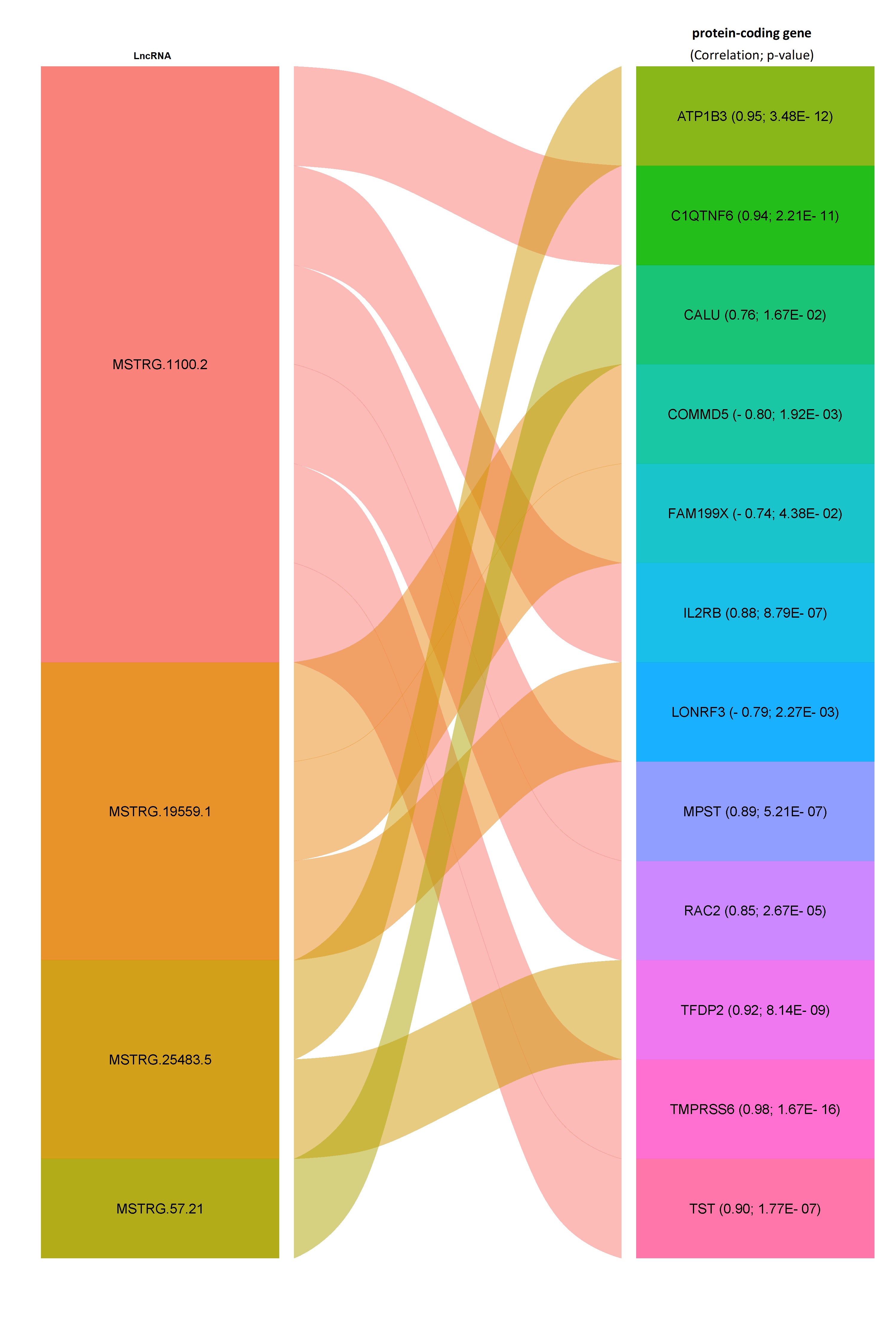


Figure S1: Alluvial plot illustrating the regulatory paths among the novel lncRNAs, and protein-coding genes. Four novel lncRNAs targeted their 12 neighbor protein-coding genes.


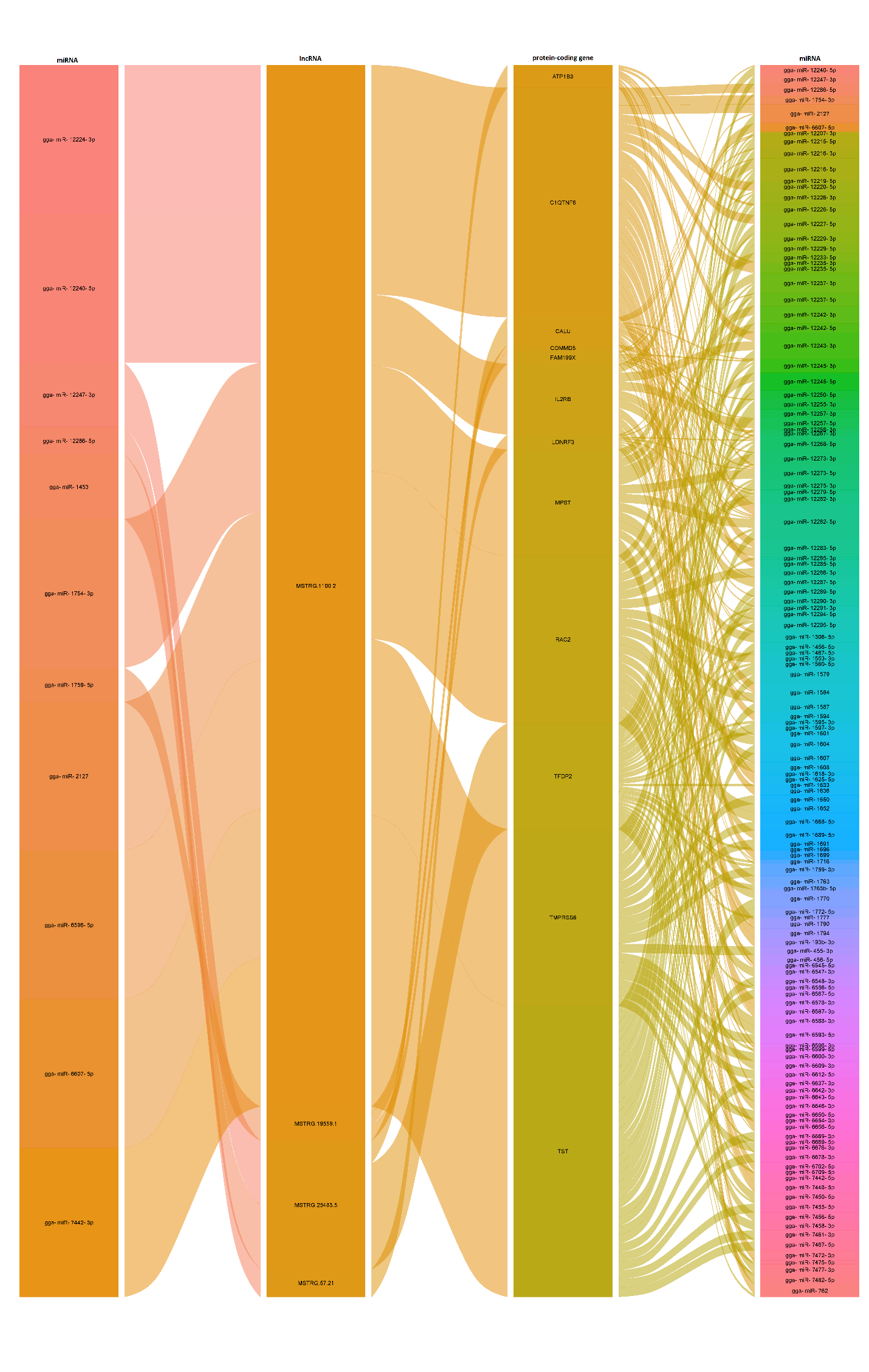


Figure S2: Alluvial plot illustrating the regulatory paths among the novel lncRNAs, miRNAs, and protein-coding genes. Four novel lncRNAs targeted their 12 neighbor protein-coding genes while were under the regulation of 11 miRNAs. A total of 128 miRNAs regulated 12 of the neighbor protein-coding genes.
